# P-873. Efficacy and Safety of Cefadroxil for Oral Transitional Therapy for Staphylococcal and Streptococcal Bloodstream Infections

**DOI:** 10.1093/ofid/ofae631.1064

**Published:** 2025-01-29

**Authors:** Stefanie Nguyen, Emi Minejima

**Affiliations:** USC Mann School of Pharmacy and Pharmaceutical Sciences; Los Angeles General Medical Center, Baldwin Park , California; Univ. of Southern California, Los Angeles, CA

## Abstract

**Background:**

Cefadroxil has a longer half-life and reduced clearance allowing for once or twice daily administration, making it an appealing alternative to cephalexin, which requires up to four times daily dosing. There has been increased interest in utilization of oral beta-lactams as oral transitional therapy for bloodstream infections (BSI) due to its favorable safety profile, however, there is currently limited data available evaluating cefadroxil for BSI.

**Methods:**

A retrospective cohort study of adult patients hospitalized with MSSA or Streptococcal BSI from January 2022 to March 2024 and transitioned from IV to oral antibiotics was conducted. Patients were grouped by cefadroxil vs other highly bioavailable antibiotics (linezolid, TMP/SMX, fluoroquinolones) and compared for clinical outcomes of 30-day all-cause mortality, 30-day readmission, and 90-day recurrence.

**Results:**

103 patients were included (cefadroxil 40 patients vs other 63 patients). Overall, 79% were male, 69% were Hispanic, 33% had uncontrolled diabetes, and median CCI was 2. Clinical presentation was similar with median Pitt bacteremia score of 0 in both groups (p=ns). 56% were MSSA BSI, and the most common *Strep* spp. was *Strep pyogenes* (15%). The most common source of infection was an intermediate-risk source of infection (78%). Median days of lead in IV antibiotic therapy did not differ between groups (cefadroxil 5 vs other 6, p=0.121). Majority of patients received cefadroxil dose of 1000 mg BID (65%). No differences were observed in 30-day all-cause mortality (cefadroxil 5% vs other 1.6%, p=0.56), 30-day readmission (30% vs 15.9%, p=0.14), or 90-day recurrence (2.5% vs 14.29%, p=0.084). Similar outcomes were seen in sub-analyses comparing cefadroxil dose, MSSA vs *Strep* spp., and source risk of BSI (p=ns). Adverse effects from oral therapy were reported in 35% of cefadroxil vs 15% in other group (p=0.12); resulting in 0 patients in cefadroxil and 4 patients in the other group changing to alternative therapy.

**Conclusion:**

Cefadroxil had similar clinical outcomes to other highly bioavailable antibiotic therapy for transitional therapy for MSSA and Streptococcal BSI. As cefadroxil can be dosed less frequently compared to certain other oral beta lactams, it may be an attractive option for outpatient therapy.

**Disclosures:**

**All Authors**: No reported disclosures

